# A Case of Glaucoma the Result of Injudicious Use of Atropia

**Published:** 1885-08

**Authors:** J. M. Hull

**Affiliations:** Augusta, Ga.; Special Professor of Diseases of Eye and Throat in the Medical College of Georgia, Medical Department University of Georgia, Etc.


### A CASE OF GLAUCOMA, THE RESULT OF INJUDICIOUS USE OF ATROPIA.

By J. M. HULL, M. D.,

SPECIAL PROFESSOR OF DISEASES OF EYE AND THROAT IN THE MEDICAL COLLEGE OF GEORGIA, MEDICAL DEPARTMENT UNIVERSITY OF GEORGIA, ETC.

Reported to the Medical Association of Georgia at its Thirty-sixth Annual Session.

There is probably no drug in our entire pharmacopœia so invaluable to the oculist as sulphate of atropia, and none more capable of doing irreparable damage when injudiciously used.

When its mydriatic effects are desired, this should be accomplished within twenty-four or thirty-six hours by the use of a sufficiently strong solution (16 grs. to ℥i; if necessary, repeated every five minutes for three times), and not by the use of weaker solutions used three times per diem through several weeks.

Nothing is to be feared from its use in this way, for if its tonic effects present themselves, they can readily be relieved by a hypodermic injection of morphia sulph., repeated if required. Its value as a remedial agent is too well known to be here commented upon, and I will simply call attention to the danger to be feared from its long-continued use.

It has been recognized for years that latent glaucoma may be awakened into an active attack by the instillation into the eye of a solution of atropia, and the recitation of the following case will, I think, establish the fact that glaucoma may be produced in a healthy eye by its injudicious use for a long time.

In October last a gentleman presented himself for treatment with the following history: In May of the same year he was struck with a piece of wood over the right cheek and brow. Owing to the brow being prominent, the eye, he says, was not at all injured. On the following day the brow, lids and cheek were black and slightly swollen. As, on the second day, the bruise looked more serious, his wife became alarmed and insisted that he should consult a physician. In order to quiet her apprehensions, he did so, and stated that the eye had not participated at all in the injury, being perfectly clear, giving no pain, and vision unimpaired.

The physician gave him a solution of atropia sulph., grs. ii to ℥i., and ordered that two or three drops be put into the eye three times a day. Maximum dilatation was produced within thirty minutes after first instillation, with all the inconvenience consequent upon paralysis of accommodation.

Upon calling on the physician the next day, he was told to bandage the eye, continue treatment, and report in a week. This he did, and despite the fact that the effects of the bruise had entirely disappeared, and there existed no signs of inflammation, he was instructed to continue the drops. This being kept up six weeks, the man noticed that, in addition to his sight having become much worse, the eye seemed to have become larger, and was perfectly hard. He presented himself to the physician, who then recognized what he had accomplished, and endeavored to rectify the damage by instillations of eserine. His efforts were, of course, attended with no success.

A careful examination elicited the following: The right (injured) eye had fixed pupil, glazed appearance, cloudy aqueous, sensibility of cornea diminished, and globe distended and hard, vision diminished to light perception. The ophthalmoscope revealed a cupped and white nerve. Vision of other eye good.

Before endeavoring to show how atropia may have brought about this result, let me ask, for a brief period, the attention of the reader, while I place before him the minute anatomy of the anterior portion of the globe. We find in the cornea, at its junction with the sclera, the circular venous sinus, called the canal of Schlemen. At this same sclero-corneal margin the iris has its attachment, and from the inner surface of the cornea minute bands run to the anterior surface of the iris, forming a number of apertures, which constitute the canal of Fontana. These apertures communicate with the canal of Schlemen, and through these channels does the aqueous humor find its way into the circulation.

Another point which must be borne in mind is, that the effete matter of the vitreous has its escape into the anterior chamber through the space between the border of the lens and ciliary processes, and thence into the circulation.

Bearing these facts well in mind, and the further fact that the essence of glaucoma is an increase in the tension of the globe, it can readily be understood how anything that can cause a closure of the space of Fontana, thereby preventing the egress of the humors of the eye, must bring about an increase in its tension, which is glaucoma.

Noyes, in his work on the diseases of the eye, says: "Within five years anatomical study has shown that an almost constant lesion in chronic glaucoma is adhesion of the periphery of the iris to the border of the sclero-corneal junction, thereby choking up the apertures of the so-called space of Fontana, leading to the canal of Schlemen."

More recently, Priestley Smith, in an extended and admirable essay, and in subsequent papers, thinks the cause of glaucoma lies in diminution of the space between the border of the lens and ciliary processes, whereby the transit of fluid from the vitreous to the anterior chamber is retarded, and the periphery of the iris is pressed into the angle of Fontana's space, and increased tension results.

Accepting these as established facts, it can readily be understood how a solution of atropia, sufficiently strong to produce and maintain maximum dilatation of the pupil, and paralysis of accommodation must, by degrees, produce an increase of tension; for the iris, when dilated *ad maximum*, is thickened at its periphery, thus compressing the space of Fontana and the space between the ciliary processes, and the border of the lens being diminished, obstructs the free flow of vitreous to the anterior chamber, and aids to force into the space of Fontana the periphery of the iris, whereby the egress of the fluids of the eye are arrested. Thus was a healthy eye destroyed by the careless use, through a number of weeks, of the most useful, and, when rightly used, harmless drug in our pharmacopœia.

